# Psychosocial Factors of Dietitians' Intentions to Adopt Shared Decision Making Behaviours: A Cross-Sectional Survey

**DOI:** 10.1371/journal.pone.0064523

**Published:** 2013-05-20

**Authors:** Sarah-Maude Deschênes, Marie-Pierre Gagnon, France Légaré, Annie Lapointe, Stéphane Turcotte, Sophie Desroches

**Affiliations:** 1 Research Centre, Centre hospitalier universitaire de Québec, Quebec City, Quebec, Canada; 2 Department of Food Sciences and Nutrition, Université Laval, Quebec City, Quebec, Canada; 3 Faculty of Nursing, Université Laval, Quebec City, Quebec, Canada; 4 Department of Family Medicine and Emergency Medicine, Université Laval, Quebec City, Quebec, Canada; University of Missouri-Kansas City, United States of America

## Abstract

**Objectives:**

While shared decision making (SDM) promotes health-related decisions that are informed, value-based and adhered to, few studies report on theory-based approaches to SDM adoption by healthcare professionals. We aimed to identify the factors influencing dietitians' intentions to adopt two SDM behaviours: 1) present dietary treatment options to patients and 2) help patients clarify their values and preferences.

**Methods:**

We conducted a cross-sectional postal survey based on the Theory of Planned Behaviour among 428 randomly selected dietitians working in clinical practice across the Province of Quebec, Canada. We performed descriptive analyses and multiple regression analyses to determine the variables that explained the variance in intention to perform the behaviours.

**Results:**

A total of 203 dietitians completed the questionnaire. Their ages were from 23 to 66 and they had been practising dietetics for 15.4±11.1 years (mean ± SD). On a scale from 1 to 7 (from strongly disagree to strongly agree), dietitians' intentions to present dietary treatment options and to clarify their patients' values and preferences were 5.00±1.14 and 5.68±0.74, respectively. Perceived behavioural control (β = 0.56, ρ<0.0001), subjective norm (β = 0.16, ρ<0.05), and moral norm (β = 0.22, ρ<0.0001), were the factors significantly predicting the intention to present dietary treatment options, while perceived behavioural control (β = 0.60, ρ<0.0001), attitude (β = 0.20, ρ<0.05), and professional norm (β = 0.22, ρ<0.001), significantly predicted the intention to help patients' clarify their values and preferences.

**Conclusion:**

Our results showed that dietitians intend to adopt the two SDM behaviours studied. Factors influencing intention were different for each behaviour, except for perceived behavioural control which was common to both behaviours. Thus, perceived behavioural control could be a key factor in interventions aiming to encourage implementation of SDM by dietitians.

## Introduction

In recent years, patients have increasingly reported a preference for sharing decisions with their healthcare professionals [1]. Shared decision making (SDM), a process designed to help healthcare professionals share decisions with patients, is a response to this preference [2]. SDM is innovative in that it integrates the principles of patient-centred care (PCC) [3] and evidence-based practice (EBP) into a single approach [4]. SDM promotes health-related decisions that are informed, value-based and to which patients adhere to [5]. The SDM process consists of a set of specific clinical behaviours that have been defined using a variety of frameworks [6]. According to a systematic review by Makoul and Clayman, the two fundamental principles common to the majority of frameworks are a) the EBP-related principle of presenting treatment options [6] based on the best evidence about the risks and benefits of all options available (including doing nothing) [4], and b) the PCC-related principle of clarifying the patient's values and preferences, i.e. what concerns them most about these options [6]. However, research shows that healthcare professionals have not, as a rule, adopted these behaviours [7] and that SDM is far from being the norm in healthcare [8], [9].

In the field of nutrition, patients' adherence to recommendations is a critical component for managing and preventing diet-related chronic disease [10]. Since presenting individualized, evidence-based dietary options and making decisions that take into account patients' values and preferences (or their specific concerns about their choices) apparently improve patient adherence to treatment [11], SDM is a promising avenue for nutrition. Although PCC and EBP are already recommended principles in standard dietetic practice [12], neither is fully integrated in clinical practice [13], [14].

### Conceptual framework

The Theory of Planned Behaviour (TPB) is the theory most frequently used to study and predict changes in healthcare professionals' behaviour [15]. The TPB [16] suggests that the intention to perform a behaviour is a factor of behaviour, and that intention, in turn, is influenced by three further factors, namely the subject's attitude toward the behaviour (positive or negative evaluation of the behaviour), subjective norm (perceived social pressure to perform the behaviour or not), and perceived behavioural control (the subject's perception of his/her ability to perform the behaviour) [17]. These three factors are based on three categories of salient beliefs: behavioural beliefs, normative beliefs and individual control beliefs, respectively [16]. Further research suggests that in addition to the factors identified by the TPB, moral norm, defined as the perceived moral correctness of a behaviour [18], and professional norm, defined as the degree to which an individual is affected by the normative pressures of his or her professional group [19], also explain the variance in healthcare professionals' intentions to perform a given behaviour [15], [20], [21], [22]. To the best of our knowledge, the factors that might contribute to predicting dietitians' intention to engage in behaviours related to SDM using the TPB have yet to be studied.

**Figure 1:**
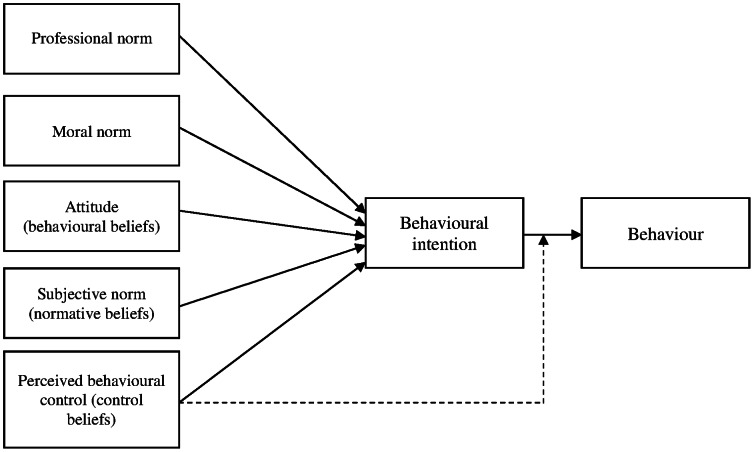
Theoretical model based on the Theory of Planned Behaviour.

The aim of our study was to use this expanded version of the TPB (Figure 1) to identify the psychosocial factors influencing dietitians' intentions to adopt the two following SDM behaviours:

1) Presenting dietary treatment options during clinical encounters with patients, and

2) Helping patients clarify their values and priorities when choosing a dietary treatment.

## Methods

### Ethics statement

Ethical approval of the project was obtained by the Research Ethics Board of the Centre hospitalier universitaire de Québec (approval S10-05-026). Participants received no remuneration for participating.

### Design, participants and recruitment

Between September 2010 and February 2011, we conducted a cross-sectional postal survey in the province of Quebec, Canada. As suggested by Godin et al. (15), a minimum of 150 participants is required to have a representative population of healthcare professionals for studies based on social cognitive theories. Considering a response rate of approximately 40% based on the literature for social-cognitive studies conducted among dietitians [21], [23], we calculated that a population of 428 dietitians was required to obtain a minimum of 150 participants with an estimated response rate of 35%. Accordingly, the Professional Order of Dietitians of Quebec (OPDQ), the professional regulatory body of dietitians, randomly selected 428 dietitians from its list of dietitians practising in clinical nutrition in hospitals across the province in 2010. We mailed dietitians a letter presenting the study, an information form, and a questionnaire. The letter informed them that returning the completed questionnaire would indicate their consent to participate in the study and that returning a non-completed questionnaire would be perceived as a refusal to participate. Inspired by the Dillman method [24], we sent three reminder letters on the second, fourth and seventh week after the first mailing.

### Data collection procedure and instrument development

We used the TBP to design a self-administered questionnaire [25] comprising 71 questions separated into three sections. The first two sections contained questions evaluating respondents' intention to adopt the two SDM-related behaviours during clinical encounters: 1) presenting patients with evidence-based dietary treatment options for their health condition, and 2) helping patients clarify their values and preferences when choosing a dietary treatment. These two SDM-related behaviours were selected according to a systematic review by Makoul and Clayman [6] that reported that these two behaviours were included in the majority of the SDM definitions. Both sections began with a brief presentation of the behaviour and the kind of conversation that it might provoke. To prevent a possible fatigue effect that we encountered during the validation of the questionnaire, we created two versions of the final questionnaire: one with questions on behaviour 1 first, and the other with questions on behaviour 2 first. The participants received one of two versions of the questionnaire. The third section of the questionnaire solicited sociodemographic characteristics and clinical specialty. For each behaviour we developed TPB-based questions to evaluate dietitians' intention (3 questions), attitude (4 questions) and behavioural beliefs (3 questions), subjective norm (3 questions) and normative beliefs (4–5 questions), perceived behavioural control (2–3 questions) and control beliefs (5 questions) [26], [27]. Moral norm (3 questions) and professional norm (3 questions) were added to the questionnaire as both of them were previously shown to significantly influence intention of dietitians to perform a behaviour, when added to the TPB [15], [20], [21], [22]. The response format was a seven point bipolar Likert scale from strongly disagree to strongly agree.

The questionnaire was in French and was validated with five dietitians to confirm the clarity of the questions. We then proceeded with a test–retest validation to evaluate the internal consistency and the temporal stability of the questionnaire. We assessed internal consistency by means of Cronbach's alpha and the values varied between 0.71 and 0.96 for both behaviours. Values of Cronbach's alpha above 0.70 indicate a good consistency [28], [29]. We assessed temporal stability by means of intraclass correlation coefficient (ICC) and values varied between 0.44 and 0.95. According to the literature, we considered the test-retest reliability to be fair for values of ICC between 0.40 and 0.59, good for values between 0.60 and 0.74, and excellent for values higher than 0.75 [30], [31], [32]. Problematic questions were deleted and a final version of the questionnaire was prepared to be used in the present study. We also performed an exploratory factor analysis on the intentions associated with each behaviour to assess whether the two SDM behaviours were truly distinct. The exploratory factor analysis between the intentions of the two behaviours confirmed that all questions used to measure dietitians' intention to present dietary treatment options were associated with factor 1 and all questions used to measure dietitians' intention to help patient clarify their values and priorities were associated with factor 2 (Table 1).

**Table 1:** Factor analysis of intention's questions for both behaviours.

Variables	Factor 1	Factor 2
**Behaviour 1**		
**Intention to present all dietary options**		
I intend to (behaviour) … Unlikely-Likely	**0.93** *	**0.17**
I estimate that my chances to (behaviour) are… Low-High	**0.93**	**0.14**
I will (behaviour) … Disagree-Agree	**0.91**	**0.15**
**Behaviour 2**		
**Intention to clarify values and preferences**		
I intend to (behaviour) … Unlikely-Likely	**0.17**	**0.88**
I estimate that my chances of (behaviour) are… Low-High	**0.15**	**0.86**
I will (behaviour) … Disagree-Agree	**0.11**	**0.81**

*Factor loading (the correlation coefficient between the variables and the factors).

To prevent possible contamination of the population by our recruiting only those working in hospitals, we validated our questionnaire with randomly selected dietitians counselling clients in private or community settings.

### Data analyses

We calculated descriptive statistics with the theoretical factors' questions and the sociodemographic questions. We performed factor analysis with an acceptable value of Kaiser's Measure of Psychometric Sampling Adequacy. We performed factor extraction on the results for eigenvalues and a varimax rotation. We considered an item's factor loading of higher than 0.4 to be good. The association between factors and intention items could be observed with high item factor loading for the first behaviour and item loading near to 0 for the other behaviour. In this case, the orthogonality of the two behaviours was verified. We also performed t-tests between intentions for both behaviours to determine if there was a significant difference between the behaviours themselves. We performed a Pearson correlation between the intentions for the two behaviours to verify the relationship. To determine the theoretical variables that best explained the variance in dietitians' intentions to perform the behaviours, we performed multiple linear regression analyses. Whenever necessary, we adjusted for potential confounding factors found with bivariate analyses. To find potential confounding factors, we performed ANOVAs or t-tests between factors and the intention considering a p value <0.10. If confounding factors were found, they were included in the multiple regressions. With a view to carry out an intervention to promote the practice of SDM in nutrition, it is suggested to identify the underlying beliefs of the intention [33]. Therefore, we performed multiple linear regressions using the salient beliefs underlying each construct that had been found significant in influencing the intention to perform each behaviour. The level of significance was established at less than p<0.05. All statistical analyses were performed with SAS version 9.2 (SAS Institute Inc., Cary, N.C., USA).

## Results

### Participants' characteristics

**Figure 2:**
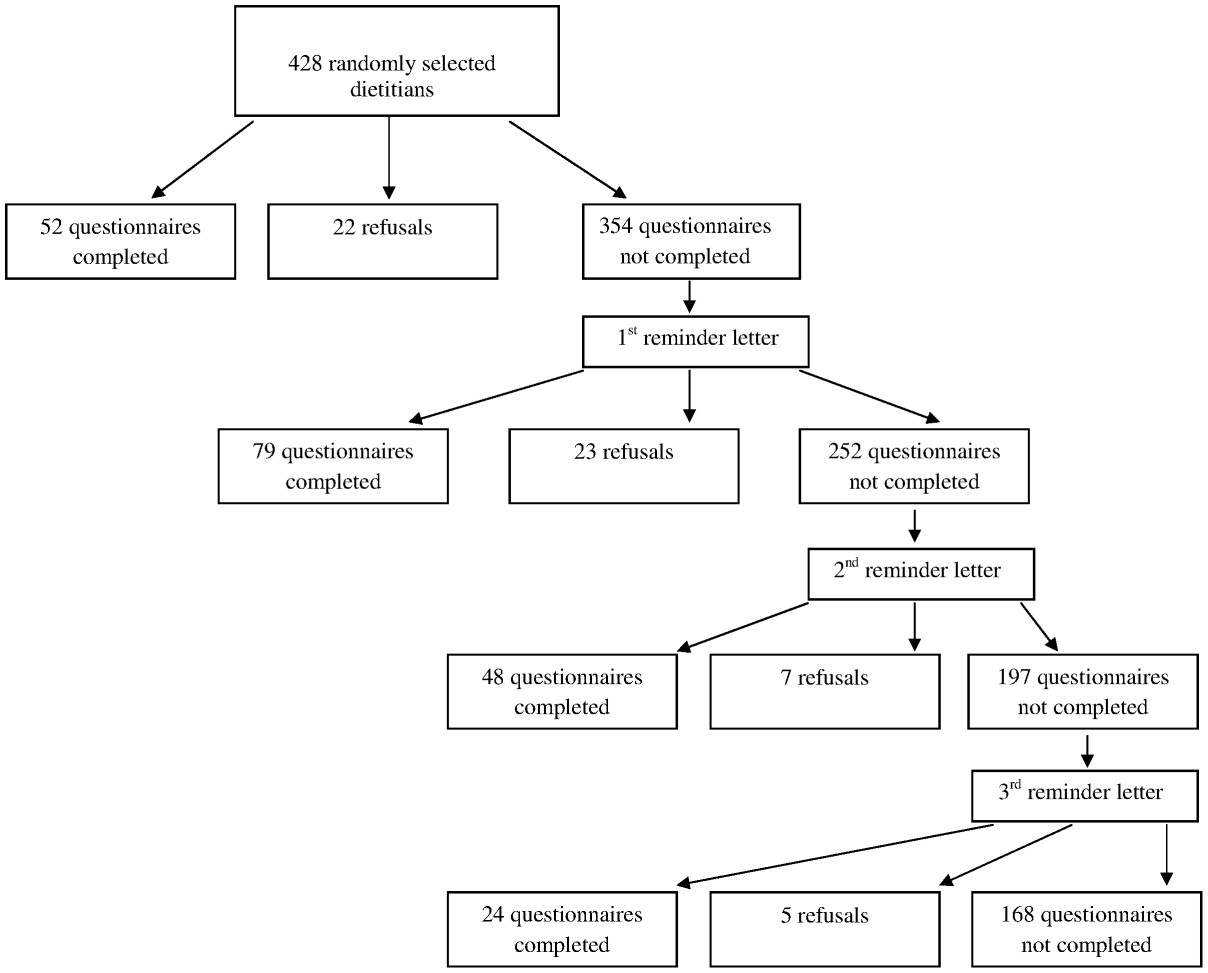
Flow chart of study participants.

Of the 428 questionnaires sent out, 203 were completed and returned (response rate 47%). Fifty-seven potential participants expressed a refusal to participate in our study by returning a non-completed questionnaire and 168 people did not return their questionnaires (Figure 2). Sociodemographic characteristics of participants are presented in Table 2.

**Table 2:** Sociodemographic characteristics of participants.

Characteristics	
**Age (years) (N = 203)**	**40.3±10.9** *
**Years in practice (N = 202)**	**15.4±11.1** *
**Gender (N = 203)**	
Female	**195 (96.0)** †
Male	**8 (4.0)** †
**Works within multidisciplinary team (N = 202)**	**190 (94.0)** †
**Region (N = 201)**	
Montreal	**68 (33.8)** †
Quebec City	**40 (19.9)** †
Monteregie	**22 (10.9)** †
Other regions	**71 (35.3)** †

*Data in mean ± SD

† Data in number (%)

### Descriptive analysis of theoretical factors

The intention to perform both behaviours was relatively high (mean of 5.00±1.14 for behaviour 1 and mean of 5.68±0.74 for behaviour 2) (Table 3), but dietitians reported a lower intention to present dietary treatment options (behaviour 1) than to help their patients clarify their values and preferences (behaviour 2) (t = −8.29, *P*<0.0001). The intentions of the two behaviours were significantly correlated (r = 0.37, *P*<0.0001).

**Table 3:** Descriptive statistics of the theoretical factors for both behaviours.

Behaviour 1To present dietary treatment options during clinical encounters with patients			Behaviour 2To help patients clarify their values and preferences when choosing a dietary treatment	
Variable	Mean	SD	Mean	SD
**Intention**	**5.00**	**1.14**	**5.68**	**0.74**
**Attitude**	**4.86**	**0.89**	**5.30**	**0.76**
**Behavioural beliefs**	**5.59**	**0.93**	**6.07**	**0.65**
**Subjective norm**	**5.14**	**1.02**	**5.55**	**0.80**
**Normative beliefs**	**5.30**	**0.96**	**5.75**	**0.74**
**Perceived behavioural control**	**4.86**	**1.24**	**5.34**	**0.75**
**Control beliefs**	**4.43**	**1.20**	**5.44**	**0.78**
**Moral norm**	**4.66**	**1.22**	**5.40**	**0.89**
**Professional norm**	**5.75**	**0.75**	**6.04**	**0.67**

### Psychosocial factors and their influence on intentions

Table 4 shows the results of the multiple linear regressions on the intention to present dietary treatment options (behaviour 1). The regression model explained 85% of the variance of the intention. The main factors influencing the intention to present dietary treatment options were perceived behavioural control (β = 0.56, *P*<0.0001), moral norm (β = 0.22, *P*<0.0001) and subjective norm (β = 0.16, *P* = 0.05). The salient beliefs that were significant for this intention were the patient's family support (β = 0.37, *P*<0.0001), lack of time (β = 0.20, *P*<0.0001), the multidisciplinary team (β = 0.13, *P* = 0.02) and lack of interviewing skills (β = 0.12, *P* = 0.005). The regression model including the beliefs explained 82% of the variance of the intention. Table 5 shows the results of the multiple regressions on the intention to help patient clarify their values and preferences (behaviour 2). The model explained 77% of the variance in intention. The factors that significantly explained the intention to help patients clarify their values and preferences were perceived behavioural control (β = 0.60, *P*<0.0001), professional norm (β = 0.22, *P* = 0.0005) and attitude (β = 0.20, *P* = 0.004). Salient beliefs which significantly explained the intention to perform this behaviour were lack of time (β = 0.28, *P*<0.0001), the fact that it helps to tailor the treatment (β = 0.19, *P* = 0.0003), the patient's level of motivation (β = 0.14, *P*<0.002) and lack of interviewing skills (β = 0.09, *P* = 0.006). The regression model with the beliefs explained 74% of the variance of the intention. Finally, age, years of practice, sex and the level of education were not statistically significant in explaining the intention of adopting any of the two behaviours.

**Table 4:** Factors influencing the intention to present dietary treatment options during clinical encounters with patients (behaviour 1).

Variables	Coefficient β	P-value
**Theoretical factors**		
**Attitude**	**0.004**	**0.55**
**Subjective norms**	**0.16**	**0.01**
**Perceived behavioural control**	**0.56**	**<0.0001**
**Moral norms**	**0.22**	**<0.0001**
**Professional norms**	**0.10**	**0.14**
**Beliefs**		
**Normative beliefs**		
Other dietitians	**0.06**	**0.11**
The physician	**−0.01**	**0.82**
The patient	**0.01**	**0.83**
The patient's family	**−0.06**	**0.28**
The multidisciplinary team	**0.13**	**0.02**
**Control beliefs**		
The patient's level of understanding	**0.06**	**0.07**
Lack of time	**0.20**	**<0.0001**
The patient's family support	**0.37**	**<0.0001**
Lack of interviewing skills	**0.12**	**0.005**
The patient's level of motivation	**0.06**	**0.17**

R^2^ = 0.85 (for theoretical factors) R^2^ = 0.82 (for the beliefs).

**Table 5:** Factors influencing the intention to help patients clarify their values and priorities when choosing a dietary treatment (behaviour 2).

Variables	Coefficient β	P-value
**Theoretical factors**		
**Attitude**	**0.20**	**0.004**
**Subjective norms**	**0.04**	**0.45**
**Perceived behavioural control**	**0.60**	**<0.0001**
**Moral norms**	**0.01**	**0.82**
**Professional norms**	**0.22**	**0.0005**
**Beliefs**		
**Behavioural beliefs**		
Helps to tailor the treatment	**0.19**	**0.0003**
Helps patient's adherence to treatment	**0.06**	**0.15**
Creates a trust relationship	**0.08**	**0.14**
**Control beliefs**		
Lack of time	**0.28**	**<0.0001**
Lack of interviewing skills	**0.09**	**0.006**
The patient's level of motivation	**0.14**	**0.002**
The trust between dietitian and patient	**0.06**	**0.07**
The patient's level of openness	**0.03**	**0.56**

R^2^ = 0.77 (for theoretical factors) R^2^ = 0.74 (for the beliefs).

## Discussion

To our knowledge, this is the first study to identify the psychosocial factors influencing dietitians' intentions to adopt two behaviours fundamental to engage in SDM. Our results indicate that dietitians have a positive intention towards each behaviour, but that different factors predict the intention to perform each behaviour. Dietitians' intention to present dietary treatment options to patients was predicted by perceived behavioral control, subjective norm, and moral norm. Interestingly, these factors are similar to the predictors of nurses' intention to integrate research evidence into clinical decision-making [34], suggesting that EBP-related behaviours may be similar across healthcare professionals. Along with subjective norm, the normative belief found significant was “the multidisciplinary team” which revealed that dietitians may feel peer pressure to engage in this behaviour. Moral norm was also a significant predictor of the intention to present options. To engage in SDM, healthcare professionals must explain the risks and benefits of the available treatment options including the option of doing nothing [4], [35]. We hypothesize that some dietitians may feel morally dubious to discuss the option of doing nothing with their patients because they want the well-being of their patients. Another possible explanation of the importance of moral norm may reside in the difficulty healthcare professionals have in disclosing the scientific uncertainty about treatment options and their concern that it could increase the uncertainty of the patient with regard to the treatment options. This was recently found to be negatively associated with the intention to adopt SDM [36]. Our results suggest that perceived behavioural control, professional norm and attitude predict dietitians' intention to help patients clarify their values and preferences. Therefore, those designing SDM interventions for this group should consider addressing the barriers, facilitators, advantages and disadvantages that dietitians perceive with regard to performing the behaviour, as well as the pressure they feel from their fellow professionals. As clarifying values and preferences is already a standard skill in dietetic clinical practice [12], [37], it is not surprising that professional norm was found to be a significant factor influencing the intention to adopt this behaviour. The promotion of this behaviour by professional bodies combined with improving dietitians' skills in helping their patients clarify values and preferences could lead to successful implementation of this behaviour in dietetic practice. Our results show that although dietitians' intentions to perform the two behaviours were positively correlated, their intention to help patients clarify their values and preferences was stronger than their intention to present dietary treatment options to them. This may suggest that PCC-related behaviours are more integrated into dietitians' current practice than EBP-related behaviours.

Perceived behavioural control was the factor that explained the largest variance of the intention to adopt each of the behaviours. This factor is most often associated with the intention among health professionals [15]. Also, a study based on the TPB that also explored other SDM–related behaviours among healthcare professionals revealed that this factor was the most significant predictor of the intention [38]. With regard to individual control beliefs, lack of time and lack of interviewing skills were those common to both behaviours in significantly explaining intention. Regardless of the targeted behaviour, these results suggest that interventions such as training in interviewing techniques and time management would support the implementation of SDM by dietitians. Our study reinforces some of the distinguishability of the elements of SDM model. Factor analysis revealed that the questions associated with the intention to perform one behaviour were mostly different from the questions associated with the other, confirming assertions in the literature on models and definitions of SDM that these behaviours are indeed determined by distinct factors [6]. We also observed a moderate [39] but significant correlation between the intentions to adopt the two behaviours, confirming that although the behaviours are distinct, they both properly belong within the overarching SDM model. Our results therefore concur with the current state of knowledge on SDM, and suggest that the development of interventions to encourage SDM among dietitians should target predictors related to each key behaviour.

Our study has several strengths. First, dietitians who participated in our study were chosen at random and we obtained a response rate (47%) which is substantially similar to other studies using a theoretical model to predict the intention of dietitians to adopt a behaviour [21], [23]. However, we acknowledge that our sample do not represent the entire population of dietitians. Second, our population comprised entry-level dietitians as well as more experienced providers and was composed with 96% of women, which is representative of the population of dietitians in Canada [40]. Third, participants practiced in the three most populous regions in the province of Quebec, Canada. Therefore, results of the present study are likely to be representative of dietitians practicing clinical nutrition in the province of Quebec [40]. The study also had some limitations. First, it is possible that participants who responded to the questionnaire had a favourable perception of the behaviours. However, we did not find any difference in intention when comparing early responders, i.e. people who responded before we sent the first reminder letter, vs. late responders, i.e. people who responded after we sent at least one reminder letter. Moreover, we found no difference between dietitians responding before the median number of days taken to return the questionnaire vs. those who responded after the median, which strongly suggest that early responders did not have a more positive intention towards the behaviours than late responders. Second, as we did not fit the behaviours under study to a specific nutritional clinical context, our results are not associated with specific clinical situations. SDM may be more suitable for chronic health conditions where self-management is of the utmost importance, such as diabetes [5], and we cannot exclude the possibility that some participants were practising in a context in which SDM is not easily integrated or considered relevant (e.g. intensive care unit). Third, this study explored the factors influencing the intention to perform the behaviours, but we did not explore the gap between intention and behaviour, as we did not evaluate the behaviour itself in our population. Therefore, no causal inference should be made. Also, it is possible that intention was not the only factor influencing these behaviours. As proposed by the TBP, perceived behavioural control can have an impact on behaviour independent of its impact on intention [15], [16]. In this study, perceived behavioural control was the theoretical factor that best predicted dietitians' intention to adopt the two studied SDM behaviours.

## Conclusions

This is the first questionnaire to evaluate two SDM behaviours concomitantly, and to apply a theory-based approach to studying SDM among dietitians. Our results suggest that dietitians intend to adopt both SDM behaviours studied. While most factors of intention were different for each behaviour, perceived behavioural control was common to both behaviours, suggesting it could be a key ingredient in interventions aiming to encourage implementation of SDM by dietitians. These results will contribute to develop interventions to implement SDM in nutrition clinical practice using a longitudinal design.

## Supporting Information

Questionnaire S1 Self-administered questionnaire on the dietitians' intention to adopt the two SDM-related behaviours during clinical encounters based on the TPB.
(DOCX)
