# Supplementary material for: Psychosocial Factors of Dietitians' Intentions to Adopt Shared Decision Making Behaviours: A Cross-Sectional Survey
Source: PLoS One. 2013 May 20;8(5):e64523. doi: 10.1371/journal.pone.0064523 (PMC3659101; doi:10.1371/journal.pone.0064523)
Supplement: Questionnaire S1 — Self-administered questionnaire on the dietitians' intention to adopt the two SDM-related behaviours during clinical encounters based on the TPB. (DOCX) [file pone.0064523.s001.docx]

| **date** | | | **initiales du sujet** | | **# sujet (codification)** | |
| --- | --- | --- | --- | --- | --- | --- |
| **jour** | **mois** | **année** | **prénom** | **nom** | **PDID1** |  |

Questionnaire sur des comportements reliés à la prise de décision partagée

La science de la nutrition est en évolution constante et de plus en plus d’évidences scientifiques permettent d’établir un lien entre l’alimentation et la santé. De nombreuses approches nutritionnelles fondées sur des données probantes ont été identifiées, ce qui permet d’offrir à la population un nombre croissant d’options nutritionnelles pour la prévention ou la prise en charge d’une même condition de santé reliée à la nutrition. Les résultats de diverses enquêtes suggèrent qu’une vaste majorité de Canadiens et de Canadiennes désirent participer activement à la prise de décision portant sur leur santé. Ainsi, cela suggère que les patients auront besoin davantage de soutien de la part des diététistes afin de favoriser une prise de décision éclairée et une participation active dans la prise de décision en matière de traitements nutritionnels**. La prise de décision partagée est une approche qui intègre la pratique selon les données probantes et l’approche centrée sur le patient. Elle se définit comme étant un processus de prise de décision qui nécessite la participation conjointe d’un patient et d’un professionnel de la santé.** La prise de décision partagée s’appuie sur les meilleures évidences scientifiques associées aux avantages et aux inconvénients de toutes les options disponibles, et permet au patient de connaître toutes les options de traitements possibles adaptées à sa condition de santé et de choisir celle qui convient le mieux à ses valeurs et à ses préférences. Malgré l’intérêt suscité auprès de la pratique médicale, la prise de décision partagée est très peu étudiée chez d’autres professionnels de la santé et aucune étude n’a encore été effectuée dans le domaine de la nutrition.

Nous désirons connaître votre opinion sur l’adoption de deux comportements reliés à la prise de décision partagée soient : le fait de présenter à vos patients toutes les options de traitements nutritionnels appropriés à leur condition de santé lors d’une consultation (partie I) et sur le fait de clarifier les valeurs et les préférences qui sont importantes pour vos patients face aux différentes options de traitements nutritionnels lors d’une consultation (partie II).

Instructions

- Ce questionnaire est codé et seule la chercheure principale peut le relier à votre nom.
- Par souci environnemental, ce questionnaire comporte **des questions au recto et au verso des pages**.
- Inscrivez un X dans la case appropriée à votre choix de réponse.
- Il est important de répondre à toutes les questions même si elles peuvent vous sembler répétitives.
- Répondez en cochant une seule case par réponse.

Exemple :

1. Pour moi, faire de l’artisanat c’est…

*Si vous pensez que faire de l’artisanat est assez agréable vous devez l’indiquer comme suit :*

|  |  |  |  | X |  |  |
| --- | --- | --- | --- | --- | --- | --- |
| Extrêmement  désagréable | Très  désagréable | Assez  désagréable | Ni un  Ni l’autre | Assez  agréable | Très  agréable | Extrêmement  agréable |

Définition

**Personnes importantes pour vous** : Fait référence à des personnes ou groupes de personnes jugées importantes pour vous (elles peuvent être dans votre vie personnelle ou professionnelle) et dont l’opinion sur le fait d’utiliser le processus de soins en nutrition dans votre pratique est importante pour vous.

Merci de votre participation!

PARTIE I

1. Pour plusieurs conditions de santé reliées à la nutrition, différentes options de traitements sont possibles. Par exemple, pour traiter l’hypercholestérolémie, la diète méditerranéenne et la diète réduite en matières grasses ont respectivement démontré leur efficacité. Chaque traitement nutritionnel comporte des avantages et des inconvénients dont la (le) diététiste et le patient doivent tenir compte afin de prendre une décision éclairée. De plus, peu importe la condition de santé, le patient peut choisir l’option de ne rien faire à condition qu’il en connaisse les conséquences.

Selon le scénario décrit dans le paragraphe précédent, présenteriez-vous à vos patients toutes les options de traitements nutritionnels appropriés à leur condition de santé (par exemple, la diète méditerranéenne, la diète réduite en matières grasses et ne rien faire avec leurs avantages et inconvénients) lors d’une consultation?

|  |  |
| --- | --- |
| Oui | Non |

Comment qualifieriez-vous la difficulté à prendre votre décision selon le scénario décrit ci-haut?

|  |  |  |  |  |  |  |
| --- | --- | --- | --- | --- | --- | --- |
| Extrêmement  difficile | Très difficile | Assez  difficile | Ni l’un  Ni l’autre | Assez  facile | Très  facile | Extrêmement  facile |

1. **POUR MOI**, présenter à mes patients toutes les options de traitements nutritionnels appropriés à leur condition de santé lors d’une consultation serait…

(Répondez à chaque sous-question)

2.1)

|  |  |  |  |  |  |  |
| --- | --- | --- | --- | --- | --- | --- |
| Extrêmement  agréable | Très  agréable | Assez  agréable | Ni l’un  Ni l’autre | Assez  désagréable | Très  désagréable | Extrêmement  désagréable |

2.2)

|  |  |  |  |  |  |  |
| --- | --- | --- | --- | --- | --- | --- |
| Extrêmement inutile | Très  inutile | Assez  inutile | Ni l’un  ni l’autre | Assez  utile | Très  utile | Extrêmement  utile |

2.3)

|  |  |  |  |  |  |  |
| --- | --- | --- | --- | --- | --- | --- |
| Extrêmement apaisant | Très  apaisant | Assez  apaisant | Ni l’un ni l’autre | Assez  stressant | Très  stressant | Extrêmement  stressant |

2.4)

|  |  |  |  |  |  |  |
| --- | --- | --- | --- | --- | --- | --- |
| Extrêmement désavantageux | Très  désavantageux | Assez  désavantageux | Ni l’un ni l’autre | Assez  avantageux | Très  avantageux | Extrêmement  avantageux |

1. **Les autres diététistes membres de l’OPDQ** approuveraient/désapprouveraient que je présente à mes patients toutes les options de traitements nutritionnels appropriés à leur condition de santé lors d’une consultation.

|  |  |  |  |  |  |  |
| --- | --- | --- | --- | --- | --- | --- |
| Désapprouveraient  totalement | Désapprouveraient  fortement | Désapprouveraient  légèrement | Ni l’un ni l’autre | Approuveraient  légèrement | Approuveraient  fortement | Approuveraient  totalement |

1. Même **si mon patient n’avait pas un niveau de compréhension élevé**, je lui présenterais toutes les options de traitements nutritionnels appropriés à sa condition de santé lors d’une consultation.

|  |  |  |  |  |  |  |
| --- | --- | --- | --- | --- | --- | --- |
| Extrêmement  improbable | Très  improbable | Assez  improbable | Ni l’un ni l’autre | Assez  probable | Très  probable | Extrêmement  probable |

1. Si je présentais à mes patients toutes les options de traitements nutritionnels appropriés à leur condition de santé lors d’une consultation, **la plupart des personnes qui sont importantes pour moi**…

|  |  |  |  |  |  |  |
| --- | --- | --- | --- | --- | --- | --- |
| Désapprouveraient  totalement | Désapprouveraient  fortement | Désapprouveraient  légèrement | Ni l’un ni l’autre | Approuveraient  légèrement | Approuveraient  fortement | Approuveraient  totalement |

1. Il est dans mes principes de présenter à mes patients toutes les options de traitements nutritionnels appropriés à leur condition de santé lors d’une consultation.

|  |  |  |  |  |  |  |
| --- | --- | --- | --- | --- | --- | --- |
| Extrêmement  en désaccord | Très en  désaccord | Assez en désaccord | Ni l’un ni l’autre | Assez  en accord | Très en  accord | Extrêmement  en accord |

1. J’ai l’intention de présenter à mes patients toutes les options de traitements nutritionnels appropriés à leur condition de santé lors d’une consultation.

|  |  |  |  |  |  |  |
| --- | --- | --- | --- | --- | --- | --- |
| Extrêmement  improbable | Très  improbable | Assez  improbable | Ni l’un ni l’autre | Assez  probable | Très  probable | Extrêmement  probable |

1. Je considère qu’il est correct pour une (un) **diététiste travaillant en nutrition clinique** de présenter à ses patients toutes les options de traitements nutritionnels appropriés à leur condition de santé lors d’une consultation.

|  |  |  |  |  |  |  |
| --- | --- | --- | --- | --- | --- | --- |
| Extrêmement  en désaccord | Très en  désaccord | Assez en désaccord | Ni l’un ni l’autre | Assez  en accord | Très en  accord | Extrêmement  en accord |

1. J’évalue que mes chances de présenter à mes patients toutes les options de traitements nutritionnels appropriés à leur condition de santé lors d’une consultation sont…

|  |  |  |  |  |  |  |
| --- | --- | --- | --- | --- | --- | --- |
| Extrêmement  faibles | Très  faibles | Assez  faibles | Ni l’un ni l’autre | Assez  fortes | Très  fortes | Extrêmement  fortes |

1. Même **si je manquais de temps**, je présenterais à mes patients toutes les options de traitements nutritionnels appropriés à leur condition de santé lors d’une consultation.

|  |  |  |  |  |  |  |
| --- | --- | --- | --- | --- | --- | --- |
| Extrêmement  improbable | Très  improbable | Assez  improbable | Ni l’un ni l’autre | Assez  probable | Très  probable | Extrêmement  probable |

1. Je me sentirais coupable de ne pas présenter à mes patients toutes les options de traitements nutritionnels appropriés à leur condition de santé lors d’une consultation.

|  |  |  |  |  |  |  |
| --- | --- | --- | --- | --- | --- | --- |
| Extrêmement  en désaccord | Très en  désaccord | Assez en désaccord | Ni l’un ni l’autre | Assez  en accord | Très en  accord | Extrêmement  en accord |

**Si je présentais à mes patients toutes les options de traitements nutritionnels appropriés à leur condition de santé lors d’une consultation…**

1. cela permettrait au patient d’être **plus observant au traitement.**

|  |  |  |  |  |  |  |
| --- | --- | --- | --- | --- | --- | --- |
| Extrêmement  improbable | Très  improbable | Assez  improbable | Ni l’un ni l’autre | Assez  probable | Très  probable | Extrêmement  probable |

1. cela permettrait au patient de **faire un choix éclairé.**

|  |  |  |  |  |  |  |
| --- | --- | --- | --- | --- | --- | --- |
| Extrêmement improbable | Très  improbable | Assez  improbable | Ni l’un ni l’autre | Assez probable | Très  probable | Extrêmement probable |

1. cela me ferait vivre un **sentiment d’échec ou d’incompétence.**

|  |  |  |  |  |  |  |
| --- | --- | --- | --- | --- | --- | --- |
| Extrêmement  improbable | Très  improbable | Assez  improbable | Ni l’un ni l’autre | Assez probable | Très  probable | Extrêmement probable |

1. **Les personnes qui sont importantes pour moi** me recommanderaient de présenter à mes patients toutes les options de traitements nutritionnels appropriés à leur condition de santé lors d’une consultation.

|  |  |  |  |  |  |  |
| --- | --- | --- | --- | --- | --- | --- |
| Extrêmement  en désaccord | Très en  désaccord | Assez en désaccord | Ni l’un ni l’autre | Assez  en accord | Très en  accord | Extrêmement  en accord |

1. Je considère qu’il est normal pour **une personne exerçant la profession de diététiste** de présenter à ses patients toutes les options de traitements nutritionnels appropriés à leur condition de santé lors d’une consultation.

|  |  |  |  |  |  |  |
| --- | --- | --- | --- | --- | --- | --- |
| Extrêmement  en désaccord | Très en  désaccord | Assez en désaccord | Ni l’un ni l’autre | Assez  en accord | Très en  accord | Extrêmement  en accord |

1. **Pour moi**, présenter à mes patients toutes les options de traitements nutritionnels appropriés à leur condition de santé lors d’une consultation serait :

|  |  |  |  |  |  |  |
| --- | --- | --- | --- | --- | --- | --- |
| Extrêmement  difficile | Très  difficile | Assez  difficile | Ni l’un ni l’autre | Assez  facile | Très  facile | Extrêmement facile |

1. **Les médecins** approuveraient/désapprouveraient que je présente à mes patients toutes les options de traitements nutritionnels appropriés à leur condition de santé lors d’une consultation.

|  |  |  |  |  |  |  |
| --- | --- | --- | --- | --- | --- | --- |
| Désapprouveraient  totalement | Désapprouveraient  fortement | Désapprouveraient  légèrement | Ni l’un ni l’autre | Approuveraient  légèrement | Approuveraient  fortement | Approuveraient  totalement |

1. Même **si mon patient n’avait pas de soutien de sa famille**, je lui présenterais toutes les options de traitements nutritionnels appropriés à sa condition de santé lors d’une consultation.

|  |  |  |  |  |  |  |
| --- | --- | --- | --- | --- | --- | --- |
| Extrêmement  improbable | Très  improbable | Assez  improbable | Ni l’un ni l’autre | Assez  probable | Très  probable | Extrêmement  probable |

1. Je présenterai à mes patients toutes les options de traitements nutritionnels appropriés à leur condition de santé lors d’une consultation.

|  |  |  |  |  |  |  |
| --- | --- | --- | --- | --- | --- | --- |
| Extrêmement en désaccord | Très en  désaccord | Assez en désaccord | Ni l’un ni l’autre | Assez  en accord | Très en  accord | Extrêmement en accord |

1. **Mes patients** approuveraient/désapprouveraient que je leur présente toutes les options de traitements nutritionnels appropriés à leur condition de santé lors d’une consultation.

|  |  |  |  |  |  |  |
| --- | --- | --- | --- | --- | --- | --- |
| Désapprouveraient  totalement | Désapprouveraient  fortement | Désapprouveraient  légèrement | Ni l’un ni l’autre | Approuveraient  légèrement | Approuveraient  fortement | Approuveraient  totalement |

1. Je pense que ce serait moralement inacceptable de ne pas présenter à mes patients toutes les options de traitements nutritionnels appropriés à leur condition de santé lors d’une consultation.

|  |  |  |  |  |  |  |
| --- | --- | --- | --- | --- | --- | --- |
| Extrêmement  en désaccord | Très en  désaccord | Assez en désaccord | Ni l’un ni l’autre | Assez  en accord | Très en  accord | Extrêmement  en accord |

1. **La famille des patients** approuverait/désapprouverait que je présente aux patients toutes les options de traitements nutritionnels appropriés à leur condition de santé lors d’une consultation.

|  |  |  |  |  |  |  |
| --- | --- | --- | --- | --- | --- | --- |
| Désapprouverait  totalement | Désapprouverait  fortement | Désapprouverait  légèrement | Ni l’un ni l’autre | Approuverait  légèrement | Approuverait  fortement | Approuverait  totalement |

1. Même si je **manquais d’habiletés en techniques d’entrevue**, je présenterais à mes patients toutes les options de traitements nutritionnels appropriés à leur condition de santé lors d’une consultation.

|  |  |  |  |  |  |  |
| --- | --- | --- | --- | --- | --- | --- |
| Extrêmement  improbable | Très  improbable | Assez  improbable | Ni l’un ni l’autre | Assez  probable | Très  probable | Extrêmement  probable |

1. **Les personnes les plus importantes pour moi** pensent que je devrais présenter à mes patients toutes les options de traitements nutritionnels appropriés à leur condition de santé lors d’une consultation.

|  |  |  |  |  |  |  |
| --- | --- | --- | --- | --- | --- | --- |
| Extrêmement  en désaccord | Très en  désaccord | Assez en désaccord | Ni l’un ni l’autre | Assez  en accord | Très en  accord | Extrêmement  en accord |

1. Si je voulais, je pourrais facilement présenter à mes patients toutes les options de traitements nutritionnels appropriés à leur condition de santé lors d’une consultation.

|  |  |  |  |  |  |  |
| --- | --- | --- | --- | --- | --- | --- |
| Extrêmement improbable | Très  improbable | Assez  improbable | Ni l’un ni l’autre | Assez  probable | Très  probable | Extrêmement probable |

1. Même si le **niveau de motivation de mon patient n’était pas élevé**, je présenterais à ce patient toutes les options de traitements nutritionnels appropriés à sa condition de santé lors d’une consultation.

|  |  |  |  |  |  |  |
| --- | --- | --- | --- | --- | --- | --- |
| Extrêmement  improbable | Très  improbable | Assez  improbable | Ni l’un ni l’autre | Assez  probable | Très  probable | Extrêmement  probable |

1. **L’équipe multidisciplinaire (ou interdisciplinaire)** approuverait/désapprouverait que je présente à mes patients toutes les options de traitements nutritionnels appropriés à leur condition de santé lors d’une consultation. *(Veuillez cocher «non applicable» si cette affirmation ne s’applique pas à votre milieu de travail.)*

|  |  |  |  |  |  |  |
| --- | --- | --- | --- | --- | --- | --- |
| Désapprouverait  totalement | Désapprouverait  fortement | Désapprouverait  légèrement | Ni l’un ni l’autre | Approuverait  légèrement | Approuverait  fortement | Approuverait  totalement |

|  |
| --- |
| Non applicable |

1. Je considère qu’il est correct pour une (un) **diététiste de ma région** de présenter à ses patients toutes les options de traitements nutritionnels appropriés à leur condition de santé lors d’une consultation.

|  |  |  |  |  |  |  |
| --- | --- | --- | --- | --- | --- | --- |
| Extrêmement  en désaccord | Très en  désaccord | Assez en désaccord | Ni l’un ni l’autre | Assez  en accord | Très en  accord | Extrêmement  en accord |

PARTIE II

1. S’il existe plusieurs options de traitements nutritionnels pour une même condition de santé, le patient et la (le) diététiste ont la possibilité de faire un choix quant au plan de traitement qui sera proposé au patient. Connaître les préférences et les valeurs du patient peuvent ainsi les guider afin de choisir l’option la plus appropriée pour ce patient. Par exemple, savoir ce que préfère le patient selon son horaire de travail ou sa situation familiale et savoir si la santé est une valeur importante pour lui peuvent permettre à la fois au patient et à la (au) diététiste de s’orienter vers une option de traitement qui aura été choisie d’un commun accord.

Selon le scénario décrit dans le paragraphe précédent, clarifierez-vous les valeurs et les préférences qui sont importantes pour vos patients face aux différentes options de traitements nutritionnels (en questionnant le patient sur son mode de vie, ses préférences face aux options possibles et ses valeurs) lors d’une consultation?

|  |  |
| --- | --- |
| Oui | Non |

Comment qualifieriez-vous la difficulté à prendre votre décision selon le scénario décrit ci-haut?

|  |  |  |  |  |  |  |
| --- | --- | --- | --- | --- | --- | --- |
| Extrêmement  difficile | Très  difficile | Assez  difficile | Ni l’un  Ni l’autre | Assez  facile | Très  facile | Extrêmement  facile |

1. **Les personnes les plus importantes pour moi** pensent que je devrais clarifier les valeurs et les préférences qui sont importantes pour mes patients face aux différentes options de traitements nutritionnels lors d’une consultation.

|  |  |  |  |  |  |  |
| --- | --- | --- | --- | --- | --- | --- |
| Extrêmement  en désaccord | Très en  désaccord | Assez en désaccord | Ni l’un ni l’autre | Assez en accord | Très en  accord | Extrêmement  en accord |

1. Malgré mon **manque de temps**, je clarifierais les valeurs et les préférences qui sont importantes pour mes patients face aux différentes options de traitements nutritionnels lors d’une consultation.

|  |  |  |  |  |  |  |
| --- | --- | --- | --- | --- | --- | --- |
| Extrêmement  improbable | Très  improbable | Assez  improbable | Ni l’un ni l’autre | Assez  probable | Très  probable | Extrêmement  probable |

1. J’ai l’intention de clarifier les valeurs et les préférences qui sont importantes pour mes patients face aux différentes options de traitements nutritionnels lors d’une consultation.

|  |  |  |  |  |  |  |
| --- | --- | --- | --- | --- | --- | --- |
| Extrêmement  improbable | Très  improbable | Assez  improbable | Ni l’un ni l’autre | Assez  probable | Très  probable | Extrêmement  probable |

1. Je considère qu’il est correct pour une (un) **diététiste travaillant en nutrition clinique** de clarifier les valeurs et les préférences qui sont importantes pour ses patients face aux différentes options de traitements nutritionnels lors d’une consultation.

|  |  |  |  |  |  |  |
| --- | --- | --- | --- | --- | --- | --- |
| Extrêmement  en désaccord | Très en  désaccord | Assez en désaccord | Ni l’un ni l’autre | Assez  en accord | Très en  accord | Extrêmement  en accord |

1. Il est dans mes principes de clarifier les valeurs et les préférences qui sont importantes pour mes patients face aux différentes options de traitements nutritionnels lors d’une consultation.

|  |  |  |  |  |  |  |
| --- | --- | --- | --- | --- | --- | --- |
| Extrêmement  en désaccord | Très en  désaccord | Assez en désaccord | Ni l’un ni l’autre | Assez  en accord | Très en  accord | Extrêmement  en accord |

1. Même **si je manquais d’habiletés en techniques d’entrevue**, je clarifierais les valeurs et les préférences qui sont importantes pour mes patients face aux différentes options de traitements nutritionnels lors d’une consultation.

|  |  |  |  |  |  |  |
| --- | --- | --- | --- | --- | --- | --- |
| Extrêmement  improbable | Très  improbable | Assez  improbable | Ni l’un ni l’autre | Assez  probable | Très  probable | Extrêmement  probable |

1. **POUR MOI**, clarifier les valeurs et les préférences qui sont importantes pour mes patients face aux différentes options de traitements nutritionnels lors d’une consultation serait…

(Répondez à chaque sous-question)

37.1)

|  |  |  |  |  |  |  |
| --- | --- | --- | --- | --- | --- | --- |
| Extrêmement  agréable | Très  agréable | Assez  agréable | Ni l’un  Ni l’autre | Assez  désagréable | Très  désagréable | Extrêmement  désagréable |

37.2)

|  |  |  |  |  |  |  |
| --- | --- | --- | --- | --- | --- | --- |
| Extrêmement apaisant | Très  apaisant | Assez  apaisant | Ni l’un ni l’autre | Assez  stressant | Très  stressant | Extrêmement  stressant |

37.3)

|  |  |  |  |  |  |  |
| --- | --- | --- | --- | --- | --- | --- |
| Extrêmement désavantageux | Très  désavantageux | Assez  désavantageux | Ni un ni l’autre | Assez  avantageux | Très  avantageux | Extrêmement  avantageux |

1. Je me sentirais coupable de ne pas clarifier les valeurs et les préférences qui sont importantes pour mes patients face aux différentes options de traitements nutritionnels lors d’une consultation.

|  |  |  |  |  |  |  |
| --- | --- | --- | --- | --- | --- | --- |
| Extrêmement  en désaccord | Très en  désaccord | Assez en désaccord | Ni l’un ni l’autre | Assez  en accord | Très en  accord | Extrêmement  en accord |

1. **L’équipe multidisciplinaire (ou interdisciplinaire)** approuverait/désapprouverait que je clarifie les valeurs et les préférences qui sont importantes pour mes patients face aux différentes options de traitements nutritionnels lors d’une consultation. (*Veuillez cocher «non applicable» si cette affirmation ne s’applique pas à votre milieu de travail.)*

|  |  |  |  |  |  |  |
| --- | --- | --- | --- | --- | --- | --- |
| Désapprouverait  totalement | Désapprouverait  fortement | Désapprouverait  légèrement | Ni l’un ni l’autre | Approuverait  légèrement | Approuverait  fortement | Approuverait  totalement |

|  |
| --- |
| Non applicable |

1. Pour moi, clarifier les valeurs et les préférences qui sont importantes pour mes patients face aux différentes options de traitements nutritionnels lors d’une consultation serait :

|  |  |  |  |  |  |  |
| --- | --- | --- | --- | --- | --- | --- |
| Extrêmement  difficile | Très  difficile | Assez  difficile | Ni l’un ni l’autre | Assez  facile | Très  facile | Extrêmement facile |

1. Je considère qu’il est normal pour **une personne exerçant la profession de diététiste** de clarifier les valeurs et les préférences qui sont importantes pour ses patients face aux différentes options de traitements nutritionnels lors d’une consultation.

|  |  |  |  |  |  |  |
| --- | --- | --- | --- | --- | --- | --- |
| Extrêmement  en désaccord | Très en  désaccord | Assez en désaccord | Ni l’un ni l’autre | Assez  en accord | Très en  accord | Extrêmement  en accord |

1. J’évalue que mes chances de clarifier les valeurs et les préférences qui sont importantes pour mes patients face aux différentes options de traitement nutritionnels lors d’une consultation sont…

|  |  |  |  |  |  |  |
| --- | --- | --- | --- | --- | --- | --- |
| Extrêmement  faibles | Très  faibles | Assez  faibles | Ni l’un ni l’autre | Assez  fortes | Très  fortes | Extrêmement  fortes |

1. Même si le **niveau de motivation de mon patient n’était pas élevé**, je clarifierais les valeurs et les préférences qui sont importantes pour mon patient face aux différentes options de traitements nutritionnels lors d’une consultation.

|  |  |  |  |  |  |  |
| --- | --- | --- | --- | --- | --- | --- |
| Extrêmement  improbable | Très  improbable | Assez  improbable | Ni l’un ni l’autre | Assez  probable | Très  probable | Extrêmement  probable |

1. **Les personnes qui sont importantes pour moi** me recommanderaient de clarifier les valeurs et les préférences qui sont importantes pour mes patients face aux différentes options de traitements nutritionnels lors d’une consultation.

|  |  |  |  |  |  |  |
| --- | --- | --- | --- | --- | --- | --- |
| Extrêmement  en désaccord | Très en  désaccord | Assez en désaccord | Ni l’un ni l’autre | Assez en accord | Très en  accord | Extrêmement  en accord |

1. Même **si le lien de confiance entre moi et le patient n’était pas établi**, je clarifierais les valeurs et les préférences qui sont importantes pour ce patient face aux différentes options de traitements nutritionnels lors d’une consultation.

|  |  |  |  |  |  |  |
| --- | --- | --- | --- | --- | --- | --- |
| Extrêmement  improbable | Très  improbable | Assez  improbable | Ni l’un ni l’autre | Assez  probable | Très  probable | Extrêmement  probable |

1. Je pense que ce serait moralement inacceptable de ne pas clarifier les valeurs et les préférences qui sont importantes pour mes patients face aux différentes options de traitements nutritionnels lors d’une consultation.

|  |  |  |  |  |  |  |
| --- | --- | --- | --- | --- | --- | --- |
| Extrêmement  en désaccord | Très en  désaccord | Assez en désaccord | Ni l’un ni l’autre | Assez  en accord | Très en  accord | Extrêmement  en accord |

1. Je clarifierai les valeurs et les préférences qui sont importantes pour mes patients face aux différentes options de traitements nutritionnels lors d’une consultation.

|  |  |  |  |  |  |  |
| --- | --- | --- | --- | --- | --- | --- |
| Extrêmement en désaccord | Très en  désaccord | Assez en désaccord | Ni l’un ni l’autre | Assez en accord | Très en  accord | Extrêmement en accord |

1. Je considère qu’il est correct pour une (un) **diététiste de ma région** de clarifier les valeurs et les préférences qui sont importantes pour ses patients face aux différentes options de traitements nutritionnels lors d’une consultation.

|  |  |  |  |  |  |  |
| --- | --- | --- | --- | --- | --- | --- |
| Extrêmement  en désaccord | Très en  désaccord | Assez en désaccord | Ni l’un ni l’autre | Assez  en accord | Très en  accord | Extrêmement  en accord |

**Si je clarifiais les valeurs et les préférences qui sont importantes pour mes patients face aux différentes options de traitements nutritionnels lors d’une consultation …**

1. cela permettrait de mieux **adapter le traitement au patient.**

|  |  |  |  |  |  |  |
| --- | --- | --- | --- | --- | --- | --- |
| Extrêmement  improbable | Très  improbable | Assez  improbable | Ni l’un ni l’autre | Assez  probable | Très  probable | Extrêmement  probable |

1. cela permettrait au patient **d’être observant au traitement.**

|  |  |  |  |  |  |  |
| --- | --- | --- | --- | --- | --- | --- |
| Extrêmement  improbable | Très  improbable | Assez  improbable | Ni l’un ni l’autre | Assez  probable | Très  probable | Extrêmement  probable |

1. cela me permettrait de **créer un lien de confiance** avec le patient.

|  |  |  |  |  |  |  |
| --- | --- | --- | --- | --- | --- | --- |
| Extrêmement  improbable | Très  improbable | Assez  improbable | Ni l’un ni l’autre | Assez  probable | Très  probable | Extrêmement  probable |

1. **Mes patients** approuveraient/désapprouveraient que je clarifie leurs valeurs et leurs préférences qui sont importantes pour eux face aux différentes options de traitements nutritionnels lors d’une consultation.

|  |  |  |  |  |  |  |
| --- | --- | --- | --- | --- | --- | --- |
| Désapprouveraient  totalement | Désapprouveraient  fortement | Désapprouveraient  légèrement | Ni l’un ni l’autre | Approuveraient  légèrement | Approuveraient  fortement | Approuveraient  totalement |

1. **La famille des patients** approuverait/désapprouverait que je clarifie les valeurs et les préférences qui sont importantes pour mes patients face aux différentes options de traitements nutritionnels lors d’une consultation.

|  |  |  |  |  |  |  |
| --- | --- | --- | --- | --- | --- | --- |
| Désapprouverait  totalement | Désapprouverait  fortement | Désapprouverait  légèrement | Ni l’un ni l’autre | Approuverait  légèrement | Approuverait  fortement | Approuverait  totalement |

1. Même **si le niveau d’ouverture de mon patient n’était pas élevé**, je clarifierais les valeurs et les préférences qui sont importantes pour ce patient face aux différentes options de traitements nutritionnels lors d’une consultation.

|  |  |  |  |  |  |  |
| --- | --- | --- | --- | --- | --- | --- |
| Extrêmement  improbable | Très  improbable | Assez  improbable | Ni l’un ni l’autre | Assez  probable | Très  probable | Extrêmement  probable |

1. Le contrôle que j’exerce sur le fait de clarifier les valeurs et les préférences qui sont importantes pour mes patients face aux différentes options de traitements nutritionnels lors d’une consultation est…

|  |  |  |  |  |  |  |
| --- | --- | --- | --- | --- | --- | --- |
| Extrêmement faible | Très  faible | Assez  faible | Ni l’un ni l’autre | Assez  fort | Très  fort | Extrêmement  fort |

1. Si je clarifiais les valeurs et les préférences qui sont importantes pour mes patients face aux différentes options de traitements nutritionnels lors d’une consultation, **la plupart des personnes qui sont importantes pour moi**…

|  |  |  |  |  |  |  |
| --- | --- | --- | --- | --- | --- | --- |
| Désapprouveraient  totalement | Désapprouveraient  fortement | Désapprouveraient  légèrement | Ni l’un ni l’autre | Approuveraient  légèrement | Approuveraient  fortement | Approuveraient  totalement |

1. **Les médecins** approuveraient/désapprouveraient que je clarifie les valeurs et les préférences qui sont importantes pour mes patients face aux différentes options de traitements nutritionnels lors d’une consultation.

|  |  |  |  |  |  |  |
| --- | --- | --- | --- | --- | --- | --- |
| Désapprouveraient  totalement | Désapprouveraient  fortement | Désapprouveraient  légèrement | Ni l’un ni l’autre | Approuveraient  légèrement | Approuveraient  fortement | Approuveraient  totalement |

1. Si je voulais, je pourrais facilement clarifier les valeurs et les préférences qui sont importantes pour mes patients face aux différentes options de traitements nutritionnels lors d’une consultation.

|  |  |  |  |  |  |  |
| --- | --- | --- | --- | --- | --- | --- |
| Extrêmement improbable | Très  improbable | Assez  improbable | Ni l’un ni l’autre | Assez  probable | Très  probable | Extrêmement probable |

Questions sociodémographiques

1. Quel est votre sexe?

|  |  |
| --- | --- |
| Femme | Homme |

1. Quelle est votre date de naissance?

__________/__________/__________

(an/mois/jour)

1. Depuis quelle année êtes-vous membre de l’Ordre professionnel des diététistes du Québec?

_______________________________

1. En plus de votre diplôme en diététique/nutrition, détenez-vous un autre diplôme universitaire?

|  |  |
| --- | --- |
| Oui | Non |

Si oui, veuillez indiquer lequel ou lesquels :

Baccalauréat, spécifiez la spécialité : ___________________________________

Maîtrise, spécifiez la spécialité : ___________________________________

Doctorat, spécifiez la spécialité : ___________________________________

Autre, spécifiez : ____________________________________________

1. Dans quel(s) contexte(s) clinique(s) pratiquez-vous généralement?

En centre hospitalier

Dans un CSSS

En clinique externe (ambulatoire)

En clinique interne (avec des patients hospitalisés)

En pratique privée

50/50 interne-externe

50/50 interne-privé

50/50 privé-externe

Autre (précisez) ________________________________

1. Quel(s) type(s) de problème de santé rencontrez-vous le plus souvent dans votre pratique?

_______________________________________________________________________________________________________________________________________________________________________________________________________________

1. Quel est votre statut d’emploi actuel?

Temps complet permanent

Temps partiel permanent

Temps partiel occasionnel

Autre (précisez) ________________________________________________

1. Dans l’établissement où vous travaillez, êtes-vous la (le) seul(e) diététiste?

|  |  |
| --- | --- |
| Oui | Non |

1. Dans l’établissement où vous travaillez, travaillez-vous avec une (des) équipe(s) multidisciplinaire(s) ou interdisciplinaire(s)?

|  |  |
| --- | --- |
| Oui | Non |

1. Dans quelle région travaillez-vous principalement?

Bas-St-Laurent

Saguenay-Lac-St-Jean

Capitale-Nationale

Mauricie

Estrie

Montréal

Outaouais

Abitibi-Témiscamingue

Côte-Nord

Nord-du-Québec

Gaspésie-Îles-de-la-Madeleine

Chaudière-Appalaches

Laval

Lanaudière

Laurentides

Montérégie

Centre-du-Québec

Hors Québec

Hors Canada

1. Veuillez indiquer votre participation à des comités ou groupes de travail au cours de la dernière année (Cochez tout ce qui s’applique) :

Comité institutionnel (i.e. hospitalier, CLSC, etc.)

Comité universitaire (i.e. départemental, facultaire, etc.)

Comité régional (i.e. comité régional du mois de la nutrition, etc.)

Comité provincial (i.e. OPDQ, etc.)

Comité national (i.e. Diététistes du Canada, etc.)

Autres (précisez) ___________

Aucun

1. Veuillez indiquer le(s) type(s) d’activités de formation professionnelle continue au(x)quel(s) vous avez participé au cours de la dernière année? (Cochez tout ce qui s’applique) :

Dans mon institution

Régionale

Provinciale

Nationale

Internationale

Autre (précisez) __________

Aucun

**Le questionnaire est maintenant terminé.**

**Merci de votre précieuse collaboration!**

Commentaires :

________________________________________________________________________________________________________________________________________________________________________________________________________________________________________________________________________________________________________________________________________________________________________________________________________________________________________________________________________________________________________________________________________________________________________________________________________________________________________________________________________________________________________________________________________________
